# Introducing Transthyretin as a Differentially Expressed Protein in Washing Subtype of Obsessive-Compulsive Disorder

**DOI:** 10.29252/NIRP.BCN.9.3.187

**Published:** 2018

**Authors:** Mona Zamanian Azodi, Mostafa Rezaei Tavirani, Afsaneh Arefi Oskouie, Vahid Mansouri, Mostafa Hamdieh, Naser Nejati, Mohsen Hamid-pour, Alireza Ahmadzadeh, Mohammad Rostami-Nejat, Majid Rezaei Tavirani, Seyed Abdolreza Mortazavi Tabatabaei

**Affiliations:** 1. Proteomics Research Center, School of Paramedical Sciences, Shahid Beheshti University of Medical Sciences, Tehran, Iran.; 2. Department of Psychology, School of Medicine, Shahid Beheshti University of Medical Sciences, Tehran, Iran.; 3. Department of Hematology and Blood Banking, School of Allied Medical Sciences, Shahid Beheshti University of Medical Sciences, Tehran, Iran.; 4. Gastroenterology and Liver Diseases Research Center, Research Institute for Gastroenterology and Liver Diseases, Shahid Beheshti University of Medical Sciences, Tehran, Iran.; 5. Department of Surgery, School of Medicine, Iran University of Medical Sciences, Tehran, Iran.

**Keywords:** Obsessive-Compulsive Disorder (OCD), Washing subtype, Fluoxetine, Transthyretin (TTR), Two-Dimensional gel Electrophoresis (2DE), PPI network analysis

## Abstract

**Introduction:**

Obsessive-Compulsive Disorder (OCD) as one of the important mental problems is valuable topic for proteomic research studies to better understand the underlying mechanisms of this disorder.

**Methods:**

In this paper, gel-based proteomic was used to investigate the proteome profile of 16 female patients with OCD, washing subtype before and after treatment with fluoxetine and comparing them with 20 healthy female controls.

**Results:**

One of the abnormally expressed protein spots in this study was introduced and examined for protein-protein interaction network analysis via Cytoscape and its plug-ins. Transthyretin (TTR) protein showed significant expression changes (fold change=1.7, P<0.05). While the expression level of TTR is significantly decreased in OCD patients before any treatments, the trend is partially normalized after treatment with fluoxetine in positive responders. Furthermore, TTR interaction profile shows that the proteins interacting with this protein may get affected as this protein expression trend changes in OCD patients.

**Conclusion:**

TTR can be considered for further studies to be validated as a potential biomarker for OCD.

## Highlights

- Transthyretin expression drops in OCD patients and fluoxetine partially compensates this reduction.

## Plain Language Summary

Obsessive-Compulsive Disorder (OCD) is a known mental disorder with several subtypes such as washing subgroup. The female patients suffer from this subtype experience a difficult life style. There is no molecular method for diagnosis of OCD. Interview is a common way to determine disorder and also patients’ follow up. In this regard, there is serious need for a diagnostic reagent for OCD. Since there are many molecules related to OCD, we tried to introduce a suitable and accessible molecule (which can be considered as a potential biomarker) that can use as a marker for OCD. One of the common used drugs in OCD patients is fluoxetine. Effect of fluoxetine on the presence of this molecular reagent was also investigated. The finding from serum of 16 patients compared with 20 healthy women by proteomic methods indicates that transthyretin (TTR) concentration drops in the patients and the used drug compensates this reduction considerably. This possible biomarker can be used as diagnostic factor or follow up marker of the OCD patients. More studies in the larger sample size by focus on TTR concentration on the blood of the patients can lead to support TTR role as a valuable biomarker.

## 1. Introduction

Obsessive-Compulsive Disorder (OCD) life-time prevalence is about 1% to 3% on the global scale. The typical manifestations of this severe condition are intrusive thoughts and repetitive behavior (Zamanian-Azodi et al., 2015). This complex disorder is known with different subtypes and presence of comorbidities. Its complexity has been studied via different approaches, including neurochemical, neuroanatomic, genetics, neuroimmunology, and animal studies. The conducted investigations support the contribution of dimensional genetic and environmental factors (Stein, 2000). OCD phenotype, therefore, can be influenced by interaction of these factors with each other.

There are many identified genes from genetic studies that are linked to OCD pathogenesis (Taylor, 2013). However, their associations lack genome-wide and (eQTLS) approval. Moreover, since complexity of OCD made its analyses difficult it in terms of clinical and molecular basis as well as identification of treatment protocols, subtype evaluation can be helpful (Miguel et al., 2005). Therefore, studying washing subtype of OCD as one of the common subtypes among Iranian women is crucial. Women with this phenotype have contamination fear that compel them to perform repetitive washing activities (Zamanian-Azodi et al., 2015). The hyperactivity of certain parts of the brain are identified for this subtype, including bilateral ventromedial prefrontal regions and right caudate nucleus (Mataix-Cols et al., 2004). There are some introduced genes with washing subtype, including ESR1, DLGAP1, HTR3A-E, and GRIN2B (Alonso et al., 2011; Li et al., 2015; Lennertz et al., 2014; Gratacós, Real, Bayés, Labad, & López-Solà, 2012). On the other hand, proteome investigation of OCD has not been performed yet. Proteomics can provide essential information related to disorder in molecular basis. In fact, proteome as the functional level of organism, can be identified through proteomics and thereby, better understand the underling mechanisms of the disorder (Safari-Alighiarloo, Rezaei Tavirani, Taghizadeh, Tabatabaei, & Namaki, 2016).

Available treatments for OCD are psychotherapy and medication prescription. The combination of these two are known as the most effective treatment. In addition, the most applied medication for OCD is Serotonin Reuptake Inhibitors (SSRIs) (Freitas, Ferreira, Correia, Portinha, & Correia, 2016). Fluoxetine is one of the most common types of SSRIs for OCD treatment (Gertsema, Reichenberg, & Ripperger-Suhler, 2016). Fluoxetine effects on human serum proteome is also detectable through proteomic evaluations. As serum is one of the accessible sources for exploring protein expression levels (Nejadi, Masti, Tavirani, & Golmohammadi, 2014; Nejadi et al., 2015), we aimed to assess proteome expression changes in OCD patients before and after treatment with fluoxetine. In this way, we could better understand the molecular changes at protein’s level in OCD patients.

## 2. Methods

### 2.1. Sample collection

#### 2.1.1. Human subjects

A total of 35 women with OCD, washing subtype and with moderate severity were selected for this study. The patients were diagnosed based on DSM-V and enrolled in our study from Taleghani Hospital, Tehran, Iran. The patients and 20 volunteer healthy controls (without any previous record of mental disorder) were demographically matched. Inclusion criteria were as follows: medication free women with washing subtype of OCD, without any types of other psychiatric disorder at the time of study as well as before the study. The patients were between 20–30 years old. They were given written informed consents prior to their sampling. Two expert physiatrists assessed the clinical symptoms before and after treatment with medication by using Yale–Brown Obsessive Compulsive Scale (Y-BOCS). The first sampling from OCD patients was prior to fluoxetine treatment. After 17 weeks of patients’ treatment with fluoxetine (20–60 mg range), the treatment resistance cases were excluded and only 16 samples were remained for further investigation. The patients that showed >35% reduction in Y-BOCS scores after the treatment were categorized as positive responders and their blood samples were taken. The proteome of the 16 positive responders before and after treatment with fluoxetine were compared with healthy control group.

### 2.2. Sample preparation

Blood samples were collected by venipuncture using gauge needle. The samples were left at room temperature for 30 min and then centrifuged two times (2000 g) at 4°C. Next, the separated serum samples were transferred to Eppendorf microtubes and kept at −80°C until further process.

### 2.3. Proteomic analysis

All the proteomics materials are purchased from GE Health Care Life Sciences^1^ and SERVA Company^2^. 2-DE Clean-Up Kit (GE Healthcare) was used for proteome extraction of our samples. Following the extraction, determination of protein concentration was done by 2-DE Quant Kit (GE Healthcare). The first step of separation Isoelectric Focusing (IEF) was based on pI. Prior to this step, IPG strips were passively rehydrated for 8 hours. The separation procedure was taken for 7.5 hours and the running condition was based on Bio-Rad Protocol. Between separation steps, equilibration is necessary to adjust the first step for the second condition. HPE Flat Top Tower (horizontal electrophoresis) using 2D HPE™ Double-Gel 12.5% Kit (Serva Company) separated proteins based on MW for about 3.5 hours. Finally, after 2-DE, SERVA HPE™ Coomassie® Staining Kit was applied for staining gels based on the SERVA Protocol. For gel analysis, gels were scanned by Bio-Rad Scanner and the calibration was set to GS-800 densitometer (Hasanzadeh, Rezaie-Tavirani, Seyyedi, & Emadi, 2015). The gel analysis was done by Progenesis SameSpots Software as multivariate statistical analysis tool analyzing gel images using alignment method. The criteria for gel analysis were as follow: 1.5-fold change (P<0.05). The whole experiment was done three times.

### 2.4. Nested pathway network analysis

Interaction analysis was performed by Cytoscape 3.4.0-Milestone 2 for Transthyretin (TTR) as one of the identified proteins in our experiment. TTR (initial protein) and its neighbor interacting proteins (enriched proteins) were assessed for interaction type and functional annotations by CluePedia application. The action type was derived from STRING Action File available in CluePedia panel. Selected actions are activation, binding, expression, inhibition, post translation modifications. Each action is assigned with a specific color. The edge score (Kappa Score) is customizable and scores (0–1) can be shown as thick and thin lines. Here, it is set to 0.5 (medium) cut off for any actions and also to show the top interactions (threshold selection=20 proteins). For functional enrichment analysis based on biological process, the threshold was set to 4 terms. In addition, these proteins can be visualized as a pathway-like view with pre-defined cellular compartments, including extracellular, plasma membrane, intracellular, nuclear membrane, nucleus, and transcription factor complex (Bindea et al., 2009).

## 3. Results

**Figure 1:**
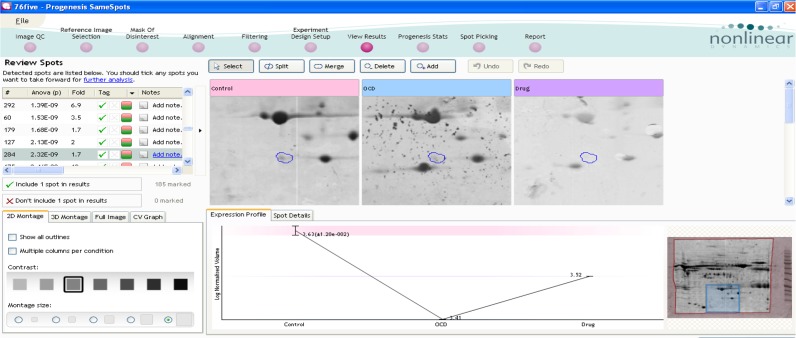
Expression pattern of the differentially expressed protein in control, OCD and drug treated samples of 2DE Gel The experiment was repeated three times by Same Spots software. The pI and MW of this spot were 5 and 1300, respectively. The left panel shows protein spot properties including spot number, P value, and fold change. Fold changes (control-OCD)=1.7 and (OCD-Drug)=1.3, P(ANOVA)=2.32E-09. Power=0.99. Q value (corrected P value)=2.32E-09. In the right corner of the image, the whole pattern of proteome profile is apparent. The red parts are not included for the alignment. The MW ladder is on the right side of the gel.

**Figure 2:**
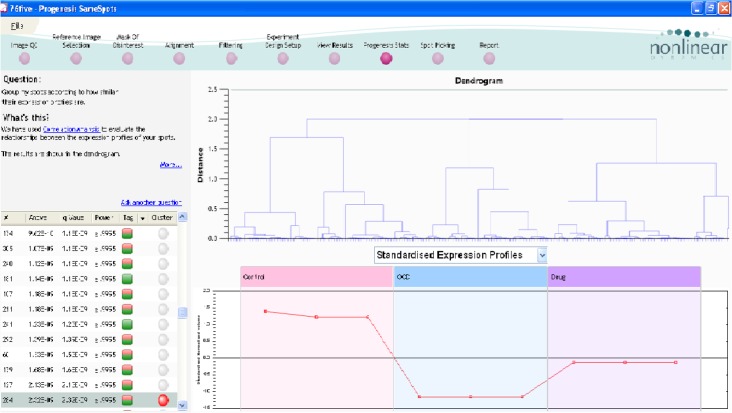
The dendrogram analysis based on hierarchical clustering and automatic correlation analysis The clustering explains expression pattern of each protein in three samples. The distance between groups implies the expression difference. TTR belongs to the second cluster. The position of this protein is highlighted in red. The vertical axis for expression pattern corresponds to the normalized volume of the spot.

**Figure 3:**
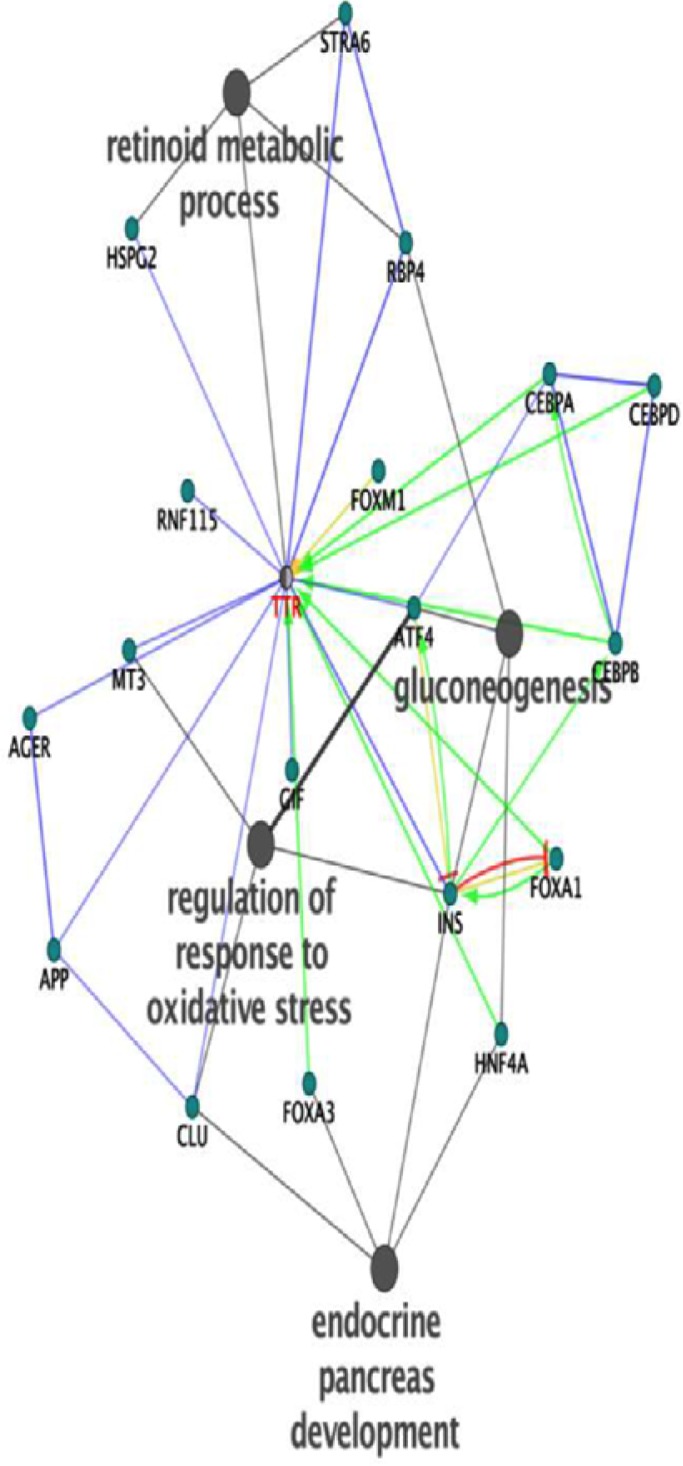
The interaction analysis of Transthyretin (TTR) with neighbor proteins and their related four top ranked biological processes (BP) colored in gray, obtained by Clue-Pedia plug-in Added proteins are colored turquoise. Different edge colors imply on different actions. Blue=Binding; Green=Activation; Yellow=Expression; Red= Inhibition

**Figure 4:**
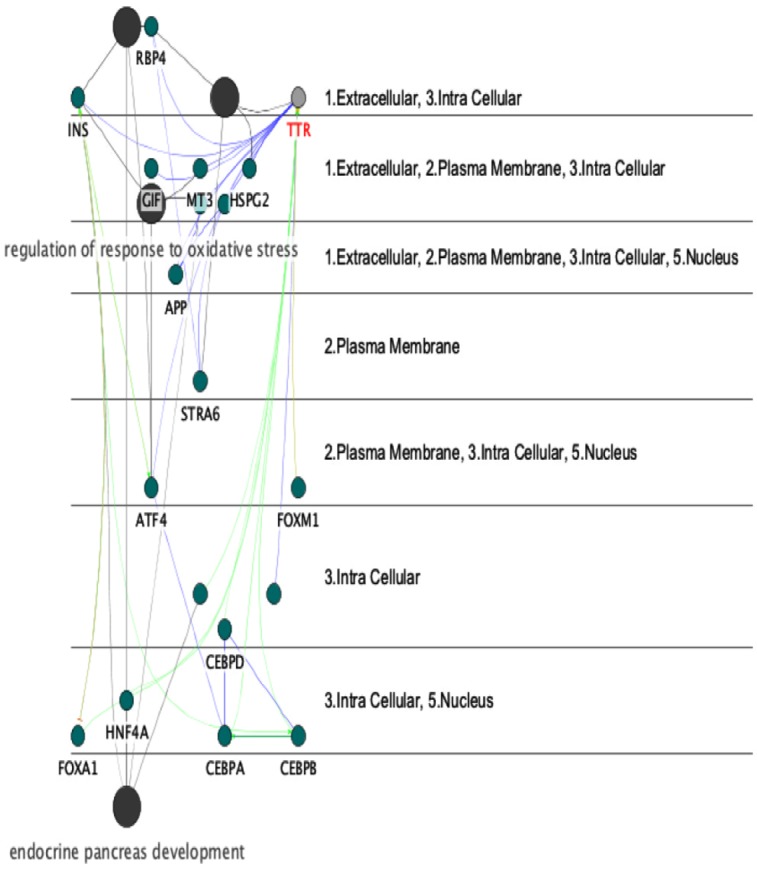
A pathway-like view of the network showing cellular locations’ of TTR and the rest of 20 proteins The location of biological processes are also clear in this Figure. These parts are coded as 1 (extracellular), 2 (plasma membrane), 3 (intracellular), and 5 (nucleus). Most of the processes are in extracellular and intracellular compartments. In addition, most of the proteins are located in intra-cellular part

The expression changes of one of the differentially expressed proteins in this study can be inferred from Figures 1 and 2. The changes range from control to drug-naïve OCD, and drug-treated samples. The interaction analysis of TTR is provided by the use of Cytoscape Plug-in, CluePedia (Figures 3 and 4).

## 4. Discussion

Performing proteomics in OCD patients before and after treatment with fluoxetine provides essential information related to proteome expression changes in these conditions. Some of the proteins with significant expression alterations may be considered as potential biomarkers after extensive validation studies. Here, among the identified spots in 2D gel electrophoresis, only one of the significant differentially expressed proteins, is examined and described. The candidate protein showed some expression levels’ changes in patients with OCD before and after treatment with fluoxetine for 17 weeks. As shown in Figure 1, this trend is decreased in drug-naïve patients while it is half way returned to its volume in treated samples. That means, the expression levels in patients with OCD before taking fluoxetine abnormally reduces and after prescription of fluoxetine for 17 weeks, this amount is half way normalized. This spot was sought against http://world-2dpage.expasy.org/swiss-2dpage/ and based on pI and MW, it was recognized as one of the isoforms of TTR protein.

Other isoforms of TTR did not show significant expression alterations in our samples. This protein is a highly conserved serum protein with many functions. It is secreted from the liver and transports thyroid hormone (T4) and Retinol-Binding Protein 4 (RBP4) and its retinol ligand. Many metabolic and septic disorders are related to dysfunction of TTR (Sullivan et al., 2006; Ingenbleek & Bernstein, 2015). In addition, TTR is also important in some brain diseases such as Alzheimer disease (White et al., 2015), schizophrenia (Yang et al., 2006), and depression (Sousa et al., 2004). It is reported that low levels of CSF-TTR has some correlation with serotonergic hypo-function in depression (Sullivan et al., 2006). In addition, TTR decrement is linked to acute phase response that could be related to inflammation (Chiaradia et al., 2012). The contribution of immune system in mental disorders including OCD (Konuk et al., 2007), is in agreement with our finding. Furthermore, as fluoxetine changes protein expression of TTR, this shows that it may have some positive regulatory effect on its levels in serum. On the other hand, it has been reported that fluoxetine impose structural changes in some proteins (Rezaei-Tavirani et al., 2011; Shahani et al., 2013). Hence, fluoxetine may have some effects on both structure and expression levels of some proteins.

Based on Figure 2, TTR belongs to the second cluster of protein expression profile. Proteins within sub-clusters may show similar annotations. Therefore, gene ontology analysis of proteins with similar expression patterns is essential for possibly identifying the role of other unknown proteins within same sub-clusters (Rezaei-Tavirani Rahmati Rad, Rezaei Tavirani, 2016). Therefore, proteins within same cluster as TTR may show similar annotations to this protein. As the expression pattern of TTR alters in OCD patients, other proteins in interaction may be influenced. In this regard, TTR protein neighbors may get affected by the dysregulation of TTR. As it is shown in Figure 3, there is no inhibitory interaction between TTR and its 20 neighbor proteins. Five activator edges directed to the TTR are highlighted in Figure 3. Therefore, TTR function is balanced in a basic state in physiologic condition and positive regulation is the main regulatory activity for evaluation of its role. Returning to the physiological condition, requires decrement of activatory regulation. FOXM1 is the unique node that affects directly TTR expression rate. Thus, expression of FOXM1 may be changed as well. Moreover, one of the important binding proteins in TTR network is insulin. Insulin was previously recognized as one of the key proteins interacting with the identified metabolites of OCD (Zamanian-Azodi, Mortazavi Tabatabaei, Mansouri, & Vafaee, 2016). This finding may provide additional validation of the possible contribution of insulin in OCD pathophysiology.

The highlighted role of insulin in this study indicated the involvement of metabolite pathways such as carbohydrate metabolism and sugar regulation in body and in OCD patients. However, regulatory role of insulin in the other biological processes implies its importance in this disorder. APP is another protein that binds to TTR and its role in mental disorders is reported (Seidel et al., 1995; Jakobsson et al., 2013). Moreover, annotation analysis showed that regulation of response to oxidative stress, gluconeogenesis, retinoid metabolic, and endocrine pancreas development are the processes related to the obtained nested-network. These processes may be altered by dysregulation of the correlated proteins; yet, more examination is required to clarify it. TTR has vast interactions with different parts of a cell as indicated in a cerebral view in Figure 4.

Proteins connected to TTR are organized in extracellular, plasma membrane, intracellular, and nucleus. In addition, intracellular part is the highlighted location for most of the retrieved interacting proteins with TTR. Moreover, TTR and INS seem to be present at the similar cellular components. This finding supports the significant roles of the two biomarkers in metabolite network. Consequently, one of the differences between normal people and patients with OCD is the expression changes of TTR protein in the human serum. As the first known proteomic study, we propose the association of TTR to the OCD pathogenicity. The findings indicate that one of the isoforms of TTR shows significant down-regulation pattern in OCD patients and can be considered as a potential biomarker after extensive validation studies. Furthermore, fluoxetine may contribute in positive regulation of TTR expression levels in serum. Similarly, for this aim, validation methods should be considered. On the whole, it can be suggested that further monitoring of TTR as a possible candidate biomarker may help better understand the molecular alteration and pathways involved in OCD patients as well as clinical response to fluoxetine.

In conclusion, proteomics introduce TTR as a significant altered protein in expression levels in OCD. Consequently, TTR may be considered as a novel serum indicator of OCD pathophysiology after implementing extensive validation approaches. In addition to TTR, it was determined that the other agents especially insulin may play crucial roles in OCD; however, the major role of TTR is highlighted.

## Ethical Considerations

### Compliance with ethical guideline

The patients were between 20–30 years old. They were given written informed consents prior to their sampling.

## Footnotes

1. http://www.gelifesciences.com

2. http://www.serva.de
